# How to Estimate the Cost of Point-of-Care CD4 Testing in Program Settings: An Example Using the Alere Pima™ Analyzer in South Africa

**DOI:** 10.1371/journal.pone.0035444

**Published:** 2012-04-20

**Authors:** Bruce Larson, Kathryn Schnippel, Buyiswa Ndibongo, Lawrence Long, Matthew P. Fox, Sydney Rosen

**Affiliations:** 1 Center for Global Health and Development, Boston University, Boston, Massachusetts, United States of America; 2 Health Economics and Epidemiology Research Office, Department of Medicine, Faculty of Health Sciences, University of the Witwatersrand, Johannesburg, South Africa; 3 Department of Epidemiology, Boston University School of Public Health, Boston, Massachusetts, United States of America; 4 Department of International Health, Boston University School of Public Health, Boston, Massachusetts, United States of America; University of Cape Town, South Africa

## Abstract

Integrating POC CD4 testing technologies into HIV counseling and testing (HCT) programs may improve post-HIV testing linkage to care and treatment. As evaluations of these technologies in program settings continue, estimates of the costs of POC CD4 tests to the service provider will be needed and estimates have begun to be reported. Without a consistent and transparent methodology, estimates of the cost per CD4 test using POC technologies are likely to be difficult to compare and may lead to erroneous conclusions about costs and cost-effectiveness. This paper provides a step-by-step approach for estimating the cost per CD4 test from a provider's perspective. As an example, the approach is applied to one specific POC technology, the Pima™ Analyzer. The costing approach is illustrated with data from a mobile HCT program in Gauteng Province of South Africa. For this program, the cost per test in 2010 was estimated at $23.76 (material costs = $8.70; labor cost per test = $7.33; and equipment, insurance, and daily quality control = $7.72). Labor and equipment costs can vary widely depending on how the program operates and the number of CD4 tests completed over time. Additional costs not included in the above analysis, for on-going training, supervision, and quality control, are likely to increase further the cost per test. The main contribution of this paper is to outline a methodology for estimating the costs of incorporating POC CD4 testing technologies into an HCT program. The details of the program setting matter significantly for the cost estimate, so that such details should be clearly documented to improve the consistency, transparency, and comparability of cost estimates.

## Introduction

In most resource-limited countries, eligibility for antiretroviral therapy (ART) for HIV/AIDS is based on a count of CD4+ T-lymphocytes. Traditional CD4 count technologies require that a venous blood sample be processed by a laboratory. After testing positive for HIV infection, patients either provide a blood sample immediately or are referred to another facility if the HIV counseling and testing (HCT) site does not take blood samples. The blood sample is then sent to a laboratory for processing. Depending on laboratory capacity, results are typically available between 2 and 14 days after the patient has provided the sample. Patients are asked to return to the clinic to receive their results, after which they are referred for future HIV care and/or treatment.

The existing literature based on data from sub-Saharan Africa suggests that an average of 40% of patients diagnosed with HIV infection either do not provide a blood sample or do not return to obtain their CD4 count results. [1], [2], [3], [4], [5], [6] One solution proposed to address this problem is the incorporation of rapid, point-of-care (POC) CD4 testing technologies into HCT programs. For example, the Pima™ Analyzer (Alere) is a rapid POC CD4 testing technology that is being validated in various settings. [7], [8], [9], [10], [11], [12] Because of the cartridge-based system and small size of the unit, it can be used in non-laboratory settings such as in mobile and fixed-site HCT programs and in antenatal clinics. A few other POC technologies are also in the development pipeline. [13]

Evidence for the impact of POC CD4 testing on linkage to HIV care and treatment after HIV testing is limited but positive. One study from Mozambique reported a reduction in total loss to follow-up before initiation of antiretroviral treatment from 64% to 33%. [14] In another study from South, the introduction of POC CD4 testing increased the proportion of ART-eligible patients (CD4 cell counts≤215) who initiated ART within 3 months of HIV testing. [15] However, for patients with higher CD4 cell counts (>215), fewer patients reported for pre-ART care. [15]

As attention shifts from validation of the POC technologies to the evaluation of their impacts on care and treatment outcomes, such as post-HCT enrollment in care and earlier initiation of ART, estimates of the costs of POC CD4 tests to the service provider will be needed for budgeting and cost-effectiveness analyses and estimates have begun to be reported. [16] The usefulness of these and future estimates will depend in large part on applying a costing methodology that is logical for the specific technology and consistently applied across countries, studies, and programs. Although methods for evaluating the costs of health interventions are well documented [17], costs typically depend on the details of the intervention and the context in which it is implemented. Without a consistent and transparent methodology, estimates of the cost per CD4 test using POC technologies, such as the Pima™ Analyzer, are likely to be difficult to compare and may lead to erroneous conclusions by program managers, funding agencies, and researchers concerning budgetary impacts and cost-effectiveness.

This paper provides a step-by-step approach for estimating the cost per CD4 test from a provider's perspective. As a concrete example, we apply this approach to one specific POC technology, the Pima™ Analyzer. This approach shows how to document explicitly the data and assumptions used to estimate the cost per test, which are needed to interpret and evaluate cost estimates. The example is illustrated with recent cost data from a mobile HCT program in Gauteng province of South Africa. [18] The paper aims to contribute to the quality of future evaluations of new POC testing technologies, such as the Pima™ Analyzer, by improving the consistency, transparency, and comparability of cost information.

## Methods

The total cost to a provider of a POC CD4 test depends on many individual pieces of information. These include the material costs per test, the salaries of staff who conduct start-up and daily quality control activities on the machine, the salaries of staff who perform the CD4 test, how the technology is integrated into patient flow and management, equipment and other related costs (e.g., insurance, replacement parts), the expected working life of the technology itself, and the opportunity cost of funds used to acquire the technology (i.e. the discount rate). To capture this information in an organized and consistent manner, the costing approach proceeds through six data input tables and associated calculations to estimate:

1. material costs per test;
2. staff time per test;
3. salary costs per test;
4. costs of daily startup and quality control;
5. equipment costs (daily equivalent costs); and
6. average number of tests completed per day when the unit is used.

The information from these 6 tables is then combined into a final table that estimates the cost of a POC CD4 test from the provider perspective. This approach can be used to evaluate the cost per test using any technology. The details of each step follow logically from the technology used as well as the program in which it is used.

The mobile program information and data used in this analysis are from a South African program implemented during 2010 in Gauteng Province, and all cost are reported in U.S. dollars (costs converted from South Africa Rand to US$ at 7.5 R/$US). This costing analysis does not involve human subjects' research. The analysis relies on readily information on various costs and basic estimates of time use to complete various tasks.

## Results

### Material costs per test (Table 1)

**Table 1:** Material costs per POC test*.

Item	Unit	US$ 2010	% of total
Gloves, powder free	Cost per test	$0.01	0.11%
Pima Finger Stick Sample Collection Kit (includes plaster, gauze, alcohol swab, Pima lancet)	Cost per test	$0.67	7.70%
Pima test cartridge	Cost per test	$7.96	91.49%
Printing CD4 results	Cost per test	$0.06	0.69%
**Total materials cost per test**	Cost per test	$8.70	100.00%

* Exchange rate = 7.5 R/$US. All costs include South Africa's value-added tax (14%). Glove cost per test based on 500 powder-free gloves for R29 (National Health Laboratory System). Sample collection kit based on price quote from Alere R501.60 per 100). Test cartridge cost based on price quote from Alere (R5,969.04 for package of 100). Printing cost per test based on R1114.92 per package of printer rolls (10 rolls per pack, 50 reports per roll from Printer documentation).

Documenting the cost of materials used for each test is the first step in the costing analysis. As outlined in Table 1 for the Pima technology, these materials include a pair of gloves, the Pima sample collection kit, the test cartridge, and paper for printing the results. All costs included South Africa VAT (14%). The total materials cost per test is $8.70, with the Pima CD4 single-use test cartridge comprising 91% of the cost.

Another type of CD4 testing technology would obviously require different types of materials so that the details of Table 1 need to be adjusted logically to be consistent with the materials needed based on the POC technology.

### Staff time per test (Table 2)

**Table 2:** Staff time per POC test.

Activity	Units	Mobile Program	Scenario A: minimal time	Scenario B: labor-intensive
Pre-test counseling (HIV testing)	minutes per patient	5	0	5
Post-test counseling (HIV testing)	minutes per patient	5	1	10
Additional counseling on POC results	minutes per patient	5	1	10
Complete POC CD4 test	minutes per patient	15	10	25
Total time	minutes per patient	30	12	50
**Staff time per POC CD4 test (hours)**	**hours per patient**	**0.50**	**0.20**	**0.83**

If POC CD4 testing is integrated into HCT programs, providing this service requires additional time of HCT staff members. How much additional staff time depends on how the testing and counseling processes are organized at the site. The information in Table 2 was based on informal interviews with staff members (nurses) of the South African mobile program. In this setting, CD4 testing is estimated to have added 5 minutes to pre-HIV test counseling when the possibility of CD4 testing was discussed with the patient, 5 minutes to post-HIV test counseling to ascertain if the patient wanted to complete a CD4 test, and 5 additional minutes of counseling after the results of the CD4 test were obtained. Table 2 also provides two other scenarios for staff time per test, with a low-end estimate of 2 additional minutes and a high-end estimate of 25 minutes.

In addition to the 15 minutes total for counseling related to the CD4 test, running the test requires staff time. For the Pima™ technology, where one test can be run at a time, the typical time per test is 30 minutes (from opening the sample collection kit through the generation of results), of which 20 minutes involves waiting for the machine to complete the test once the cartridge is inserted. If the person running the test did nothing else during these 20 minutes, a total of 45 minutes of staff time would be required for each test (15 minutes for the counseling activities and 30 minutes total for the test).

In the South African mobile program, one nurse provided all care to one patient at a time, including the POC CD4 test. Rather than doing nothing during the 20 minutes while the test was running, the nurses could typically complete other activities during a portion of this time (e.g., begin counseling on CD4 test results, complete paper work, and so on). In Table 2, an estimate of 15 minutes of staff time to complete the CD4 test (10 minutes for sampling and test preparation, and 5 minutes not otherwise engaged while the test is running).

Combining the information for each activity in Table 2, total staff time per CD4 test is estimated at 30 minutes. Table 2 also includes a low-end estimate of 12 minutes per test, which could be achieved by a program with little counseling and a staff member dedicated only to running the POC tests who can accomplish other activities while the test is running. A high-end estimate of 50 minutes per test is also included in Table 2, which could be relevant for programs with intensive counseling programs and if staff members do nothing else while waiting for the test results.

The process outlined in Table 2 can be followed to estimate the staff time per test for any type of CD4 testing technology. While the mobile program included in Table 2 used the same category of staff (nurses) for all activities, other programs would likely use different staff categories for different activities. For example, a HIV counselor might complete counseling activities, a nurse might obtain the blood sample, and a laboratory technician might run the test, and so on. For other situations, the same process as outlined in Table 2 can be followed with the appropriate adjustments.

### Salary cost per working hour and per test (Table 3)

**Table 3:** Salary cost per working hour and per test.

Type of staff member*	Units	Mobile Program (nurse paid flat daily rate)	Enrolled nurse	Staff nurse	Professional nurse	Note:
Salary (including all benefits according to SA government policy)	USD/month	NR	$1,262.18	$2,228.59	$3,492.69	
Total potential working days per year	Days/year	NR	260.00	260.00	260.00	
Annual leave	Days/year	NR	21.00	22.00	22.00	
Sick leave	Days/year	NR	10.00	10.00	10.00	
National holidays	Days/year	NR	14.00	15.00	15.00	
Actual working days per year	Days/year	NR	215.00	213.00	213.00	
Actual working days per month	Days/month	NR	17.92	17.75	17.75	
Nurse salary per actual working day	USD/day working	$117.33	$70.45	$125.55	$196.77	
Working hours per day	hours per day	8.00	8.00	8.00	8.00	
Nurse salary per working hour	**salary/hour working**	**$14.67**	**$8.81**	**$15.69**	**$24.60**	
Staff time per POC CD4 test (hours)	Hours per test (mobile program)	0.50	0.50	0.50	0.50	FromTable 2
**Nurse salary cost per test**	**Cost per test (mobile program)**	**$7.33**	**$4.40**	**$7.85**	**$12.30**	
ALTERNATIVE SCENARIOS						
Staff time per POC CD4 test (hours)	Hours per test (Scenario A)	0.20	0.20	0.20	0.20	FromTable 2
**Nurse salary cost per test**	Cost per test (Scenario A)	$2.93	$1.76	$3.14	$4.92	
Staff time per POC CD4 test (hours)	Hours per test (Scenario B)	0.83	0.83	0.83	0.83	FromTable 2
**Nurse salary cost per test**	Cost per test (Scenario B)	$12.22	$7.34	$13.08	$20.50	

Table 3 develops an estimate of the full staff salary per working hour that can be combined with the time information in Table 2 to estimate salary cost per test. The mobile program offers a very simple example because nurses were used for all patient services in the mobile program, and the nurses were hired daily at a rate $117.33 per day. With 8 working hours in a day, this translates to $14.67 per working hour and $7.33 per test in the mobile program, which required half an hour per test.

For programs in which multiple levels of staff are involved, Table 3 also provides an example using three different levels of nurses (Department of Public Service and Administration, 2011). The full cost of salary and benefits (e.g. housing allowance, taxes paid by the employer, retirement benefits, and so on), sometimes labeled “cost to company,” should be included. While there are typically 260 working days in a year, most employees actually work substantially less than this, due to holidays, annual leave, and sick leave. Nurses in the South Africa program work on average 215 days per year (17.9 days per month). With 8 working hours per day, the salary per working hour would range from $8.81 for the enrolled nurse category (average of salary range for category reported in Table 3) to $24.60 for the professional nurse category.

Using the same additional time per test as for the mobile program (0.5 hours), the salary costs per test could range from $4.40 to $12.30 depending on the level of staff used in a program. This number could fall to below $2.00 if a low-paid nurse worked in a program that completed tests very quickly, or rise to $20.50 for a high-paid nurse in a time-intensive program. In other settings and in other countries, the same processes outlined in Table 3 can be followed to estimate staff costs per test.

### Cost for daily quality control (Table 4)

**Table 4:** Costs for daily quality control.

	Units	Completed by staff nurse
Machine start up time daily (machine turned-on and controls run)	hours per day	0.5
Salary cost per hour	salary/hour working (mobile program nurse from Table 3)	$14.67
Salary cost per day for quality control when machine used	Cost per day	$7.33
Cost of “Pima Bead Standard” daily quality control (lasts for 6 months once opened)	Cost per cartridge (inc. VAT)	$41.60
Days unit used per month	Days	16
Number of working days in 6 months	Days per 6 months	96
Cost per day of Pima Bead Standard	Cost per day	$0.43
**Total cost per day for quality control**	**Cost per day (mobile program nurse)**	**$7.77**
	Cost per day (enrolled nurse)	$4.84
	Cost per day (staff nurse)	$8.28
	Cost per day (professional nurse)	$12.73

Each day a POC machine is used, it typically must be turned on and quality controls run. For the PIMA technology, a cartridge (e.g, a Pima™ Bead Standard, $41.60) is inserted into the machine, with recommended control cartridge replacement every six calendar months regardless of how often the machine is used during that time. The machine is unlikely to be used every working day in many HCT settings due to holidays, staff meetings, and so on. In Table 4 (see Table 4), 16 days per month is included, reflecting an average of four days per week when the machine is used. In this case, the total cost per day for quality control is estimated at $7.77 in the mobile program, with additional estimates based on the different levels of nurses included in Table 3.

### Equipment costs and daily equivalent (Table 5)

**Table 5:** Equipment costs*.

***Step 1. Up-Front Equipment costs***				
Testing unit	$6,688.00			
Printer	$490.50			
Bag	$252.78			
Total equipment costs	$7,431.28			
Equipment includes 1 year warranty				
After one year, annual care plan for equipment	$1,200.00			
***Step 2. Present value of equipment costs with insurance***				
Scenarios				
working life of equipment in years →	5	4	3	2
years insurance purchased →	4	3	2	1
Present value of equipment costs with insurance				
0%	$12,231	$11,031	$9,831	$8,631
**5%**	$11,686	$10,699	$9,662	$8,574
8%	$11,408	$10,523	$9,571	$8,542
***Step 3. Monthly equivalent equipment costs***				
Multiple scenarios	working life in months = = >			
Annual discount rate	60	**48**	36	24
0%	$203.85	$229.81	$273.08	$359.63
**5%**	$220.53	**$246.39**	$289.58	$376.15
8%	$231.31	$256.90	$299.92	$386.33
***Step 4. Daily equivalent equipment costs***				
Days unit used on average per month (same as in Table 4)	16			
Working life of equipment and discount rates?	working life in months = = >			
Discount rate	60	48	36	24
0%	$12.74	$14.36	$17.07	$22.48
**5%**	$13.78	**$15.40**	$18.10	$23.51
8%	$14.46	$16.06	$18.75	$24.15

If POC CD4 testing is to be offered, the technology needs to be acquired by the program. How the technology is acquired, and under what conditions, must be considered. For example, the price to purchase a Pima™ Analyzer, the Pima™ Printer, and the carrying bag was quoted at $7,431.28, which includes 14% VAT. A one-year warranty is provided by the manufacturer on the equipment. While the Pima requires no maintenance by the purchaser, the unit has parts that may require replacement over time (the battery, hinges on door, PC boards, display screen). The unit needs to be returned to Alere for such repairs. An insurance plan can be purchased from $1200 per year from Alere to cover such costs or failures after the warranty period. For this analysis, we assume that the program purchases the insurance. The issue is how to translate the up-front costs and additional insurance costs incurred in the future into a daily equivalent cost (and eventually a cost-per test completed).

Table 5 shows how to estimate the costs of equipment costs and insurance into an equivalent amount per day that the equipment is actually used. To begin, two issues need to be addressed: the working life of the equipment and the discount rate used for comparing costs over time.

Regarding the life of the equipment, relatively little information exists on the working life of new equipment under operational conditions. The machine could stolen, dropped and broken, or get wet, all of which are real possibilities especially in mobile HCT programs. For a new technology, even the average working life under good conditions is uncertain.

Regarding the discount rate, numerous texts are devoted to the appropriate discount rate to use when evaluating investment projects in the private and public sectors. [19] In South Africa in 2010, the prime interest rate for borrowing (best borrowers) was approximately 10%, with inflation at 5%. An annual real interest rate (nominal minus inflation) of 5% would be relevant for financially solid programs that could borrow to purchase the equipment. As a rough approximation, an annual rate of 5% can be converted to a monthly rate of 5%/12 = 0.4167%. A more precise calculation can be found in standard texts and online (e.g., http://www.stoozing.com/mon2yr.htm), which yield 0.407%. In most program evaluation situations, the simple conversion approach is more than adequate.

Based on up-front equipment costs of $7,341 and the$1,200 insurance plan beyond the warranty period, Table 5 (Step 2) summarizes the present value of equipment costs depending on the working life of the equipment and the discount rate. For example, if the equipment lasts for 4 years, the program purchases insurance for 3 years beyond the warranty period, and a 0% discount rate is used, the present value of these costs over the 4 years is simply $7,341+$1,200+$1,200+$1,200 = $11,031. With discounting, for example using a 5% rate, the present value of costs are somewhat lower ($7,341+$1,200/(1.05)+$1,200/(1.05)^2^+$1,200/1.05)^3^ = $10,699). This analysis uses standard procedures of calculating the present value of a stream of costs over time. [19]

For this analysis, we assume that the insurance plan covers any needed repairs. If an insurance plan is not available or an organization decided not to purchase such a plan, the likely costs of repairs and maintenance over time would need to be included nonetheless into the analysis.

Table 5, Step 3, provides a range of monthly equivalent costs for the Pima equipment with different assumptions about working life (60-24 months), the discount rate (0–8%), and the associated present value of equipment costs from Step 2. Monthly costs could be as low as $203 if the program assumed money was free (0% discount rate) and the technology lasted for 5 years. The costs would be over $350 per month if the technology lasted for 2 years (or perhaps was stolen after two years). The “pmt” function in Excel is easy to use for this step in the analysis. For example, with total equipment costs of $10,699 assuming a 4 year working and 5% annual discount rate, the payment function is coded as = pmt(0.05/12, 48, $10,699, 0, 0), which yields $246.39.

Table 5, Step 4, then converts these monthly costs into an equivalent cost per day that the equipment is used. For the mobile program included in this example, the site typically operates mobile HCT activities 4 days per week (16 days per month). Assuming as a base case that the working life is 4 years (48 months) and a 5% annual discount rate, $15.40 is the estimated daily cost of the up-front equipment purchase along with the annual insurance plan.

### Number of tests per day and per year (Table 6)

**Table 6:** Number of tests completed per day and per year.

	Tests per day	Working days per year	Test per year
Mobile progam	3	192	576
**Other Testing Scenarios**			
Scenario T1	5	192	960
Scenario T2	10	192	1920
Scenario T3	15	192	2880

Note: Days per month from Table 4.

Once the daily cost of the equipment has been calculated (Table 5), the number of tests completed on average per day is required to estimate the equipment cost per test. Table 6 includes assumptions on the number of tests run per day (3, 5, 10, and 15).

In the case of the Pima technology, which can only process one test at a time, an average of 30 minutes is needed from start (preparing to collect blood through a finger prick) to finish per test, and approximately 30 minutes to start the machine and run quality controls daily. In an eight-hour day, the theoretical maximum number of tests that could be completed with one machine in an eight-hour working day is 15. With 4 days per week and 52 weeks per year, an annual theoretical maximum per machine is 2880 tests in the mobile program. Due to holidays, a realistic maximum would be less.

Most mobile sites, as well as fixed HCT programs, would typically perform fewer than 15 tests per day as a long term average due to inconsistent patient flow. When the Pima™ machine was used in the mobile program, 3 tests per day per machine was typical.

### Total cost per test (Table 7)

**Table 7:** Full cost per POC test*.

	Mobile program	Scenario T1	Scenario T2	Scenario T3	Notes
Tests per day from Table 6	3	5	10	15	Table 6
Tests per year	576	960	1920	2880	Table 6
Total materials cost per test	$8.70	$8.70	$8.70	$8.70	Table 1
Nurse salary cost per test (mobile program nurse)	$7.33	$7.33	$7.33	$7.33	Table 3
Quality control cost per test	$2.59	$1.55	$0.78	$0.52	Table 4 (mobile program nurse)
Equipment cost and insurance per test (48 months, 5% annual discount)	$5.13	$3.08	$1.54	$1.03	Table 5
**Total cost per test**	**$23.76**	**$20.67**	**$18.35**	**$17.58**	
Failure rate	0.03	0.03	0.03	0.03	
Total cost per successfully completed test	$24.49	$21.31	$18.92	$18.12	

* Assumes the 3% of failures are not retested or full labor costs are incurred for repeating test. Minor labor savings are possible with repeat testing immediately after the failure.

Using the data, assumptions, and calculations in Tables 1, 2, 3, 4, 5, and 6, Table 7 provides a summary of the financial costs to the program providing the POC test. In Table 7, the total cost per test for the mobile program is estimated at $23.76. All of the assumptions and information included in this estimate are contained in Tables 1, 2, 3, 4, 5, and 6.

Because one test can be run at a time with the Pima machine, the resulting daily capacity constraint implies that there is little room for economies of scale to reduce the cost per test. Moving from 3 to 5 tests per day on average would reduce the cost per test to $20.67. With 15 tests per day on average, which is unlikely to be achieved in a mobile program, the cost would fall to $17.58.

In Table 7, one final piece of information is needed. Based on experience in the mobile program, some failure rate needs to be included in the analysis. In the case of the Pima technology, the machine provides an error code if the CD4 test is not successfully completed, but the reason for the error may not be clear. Possibilities include not obtaining an adequate blood sample as well as shaking of the equipment (e.g., when used inside a mobile testing van and staff are moving around in the van while a sample is processing). In Table 7, when a 3% failure rate is included in the analysis, based on information from the mobile program, the costs per test increase slightly. Results from a recent multisite evaluation report a 14% failure rate using finger-prick blood. [11]

## Discussion

As studies continue to assess the usefulness of POC CD4 testing for improving patient care and strengthening linkage to care and treatment after HIV testing, the cost of such tests will need to be estimated in a range of settings, including mobile HCT programs, fixed-site HCT programs attached to medical facilities, and those not attached to a medical facility (e.g. youth centers). These programs will need to estimate for their own situations the costs of incorporating POC CD4 testing into their services and how to adjust patient management and responsibilities of staff members to provide the service with acceptable quality and costs.

In the example developed here, we estimated that the average cost per CD4 test performed by the mobile HCT program using the Pima Analyzer was $23.76. In comparison, the fee for laboratory-based testing is around $7–8 per test. [12]

The first key message from this paper is that context matters, and therefore the detailed assumptions of any costing analysis needed to be reported along with ‘results’. The estimates of the cost of POC CD4 testing, even with the same technology such as the Pima Analyzer, will vary widely across countries (due to variations in full labor costs) and within countries due to program structure (types of staff employed, time spent with patients).

A second key message is that the cost per test is driven by two components: material costs per test and labor costs per test. Individual HCT programs have little ability to adjust material costs per test except through direct negotiation with the manufacturer. As with pharmaceuticals, it is likely that the costs of the cartridges in the case of the Pima technology (or reagents in the case of other POC testing technologies) could fall as the size of the market expands and bulk purchasing strategies are pursued.

Specific programs can manage their labor costs per test. In the South African mobile program used as an example throughout this analysis, nurses provided all HCT services (counseling, HIV testing, CD4 testing during the piloting of the POC technology, and so on). These nurses were paid an appropriate market wage in the South Africa labor market ($14.67 per hour), and the estimated labor cost per test in the mobile problem was $7.33. This labor cost is far above the $0.16–$1.15 labor cost per test recently report for a generic analysis. [20] To achieve $0.20 per test, a program would need to pay labor $1.00 per hour, below the minimum wage in South Africa, and complete all activities within 12 minutes.

Table 3 shows how the labor time per test and the wage rate interact to create the labor costs per test. Low wage labor is clearly one approach for keeping the costs of POC CD4 testing low. Whether low wage and, therefore, relatively unskilled labor can be used while maintaining high quality standards is yet to be seen. Given recent analysis showing the importance of quality sample collection for POC CD4 testing, [9] the ability to complete a valid test quickly with low paid and, therefore, low skilled labor in South Africa is questionable. One potential solution would be to provide additional training and oversight for such staff. The additional costs for training and oversight would then be included in the analysis. In other countries with significantly lower wages than in South Africa, it may be easier to keep labor costs low and test quality high.

The analysis in this paper focuses on costs per test from a provider's perspective (an HIV counseling and testing program). Three other sets of costs have not been included in this analysis. First, in certain situations and countries, POC CD4 test results will need to be integrated into existing electronic patient databases. The additional health system costs of this integration, which might include systems to scan patient IDs and results and transferring data into databases managed at other locations.

Second, if POC CD4 testing is integrated into HCT programs, general systems for staff training, quality control, and monitoring and evaluation would likely need to be developed. The structure of such systems and their costs, perhaps related to or modeled on other quality assessment programs such as the African Regional External Quality Assessment Scheme and the costs to sites of participation, are not included in this analysis [21].

And third, as with most new service provided by organizations, additional costs would be incurred to set up and integrate the service into the program, such as procuring supplies, space to store supplies and the equipment (nightly if used in the mobile program), charging of the testing unit, stock control, and so on. Organizations providing medical services already have stock control systems in place (some better than others). Each site would need to assess if their current systems and space would allow easy integration of this additional service into their program (including secure space for charging the unit). If relevant, losses in storage could be easily incorporated into the cost analysis.

Point-of-care CD4 testing technologies allow HIV counseling and testing programs as well as HIV care and treatment programs to *produce* a CD4 test result more quickly than using traditional technologies. We emphasize the word produce here because the result is produced by the program providing the service, and the structure of the program (especially patient management, types of staff providing patient care, and life of the technology) have major impacts on the cost to produce a CD4 test result. As programs continue to evaluate the usefulness of integrating various types of POC CD4 testing technologies into routine practice, the approach outline in this paper, with all details provided in Tables 1, 2, 3, 4, 5, 6, and 7, can be replicated and adjusted as needed for the specific technology to develop reasonable cost estimates.
